# Supplementary material for: Observation of Mermin-Wagner behavior in LaFeO3/SrTiO3 superlattices
Source: Nat Commun. 2024 Jun 22;15:5313. doi: 10.1038/s41467-024-49518-0 (PMC11192889; doi:10.1038/s41467-024-49518-0)
Supplement: Supplementary file 1 — Supplementary Information [file 41467_2024_49518_MOESM1_ESM.pdf]

# Observation of Mermin-Wagner behavior in $LaFeO_3/SrTiO_3$ superlattices

M. Kiaba,[1,*] A. Suter,[2] Z. Salman,[2] T. Prokscha,[2] B. Chen,[3] G. Koster,[4] and A. Dubroka[1,5]

[1]*Department of Condensed Matter Physics, Faculty of Science, Masaryk University, Kotlářská 2, 611 37 Brno, Czech Republic*
[2]*Laboratory for Muon-Spin Spectroscopy, Paul Scherrer Institute, 5232 Villigen PSI, Switzerland*
[3]*Key Laboratory of Polar Materials and Devices (MOE) and Department of Electronics, East China Normal University, Shanghai 200241, China*
[4]*MESA+ Institute for Nanotechnology, University of Twente, 7500 AE Enschede, The Netherlands*
[5]*Central European Institute of Technology, Brno University of Technology, 612 00 Brno, Czech Republic*

# Supplementary information

## I. SAMPLE CHARACTERIZATION

The X-ray diffraction data shown in the article Fig. 1(c) exhibit the superlattice diffraction peaks ($SL_{-1}$ and $SL_1$) of the $(LaFeO_3)_m$+$(SrTiO_3)_5$ bilayer. The thickness of the bilayer was determined from the angular position of these diffraction peaks using the Bragg equation. Supplementary Fig. 1(a) shows the obtained bilayer thickness as a function of $m$, which is in good agreement with the thickness expected from the bulk lattice constants of $SrTiO_3$, $a_{SrTiO_3} = 3.905$ Å, and the $LaFeO_3$ pseudo-cubic lattice constant $a_{LaFeO_3} = 4.002 \pm 0.002$ Å. The latter value is an out-of-plane lattice constant that was determined from X-ray diffraction of an epitaxial 8 nm thin $LaFeO_3$ film, see Supplementary Fig. 1 (b). Its pseudomorphic structure was confirmed by reciprocal space mapping around the (004) $SrTiO_3$ peak shown in Supplementary Fig. 2(a) and around the (024) $SrTiO_3$ diffraction shown in Supplementary Fig. 2(b).

To determine whether the superlattices are relaxed or pseudomorphic, we performed the X-ray diffraction reciprocal space mapping. We measured a map near the symmetrical (004) $SrTiO_3$ diffraction shown in Supplementary Fig. 3(a), (b) and (c) for superlattices with $m$ = 1, 2 and 3, respectively, and a map near the asymmetrical $SrTiO_3$ (024) diffraction shown in Supplementary Fig. 3(e), (f), (g), respectively. The substrate diffractions, the superlattice zero- and first-order diffractions are denoted in the figures. Since all superlattice diffraction peaks have the same $Q_x$ value as those of the substrate, we conclude that all superlattices are pseudomorphic.

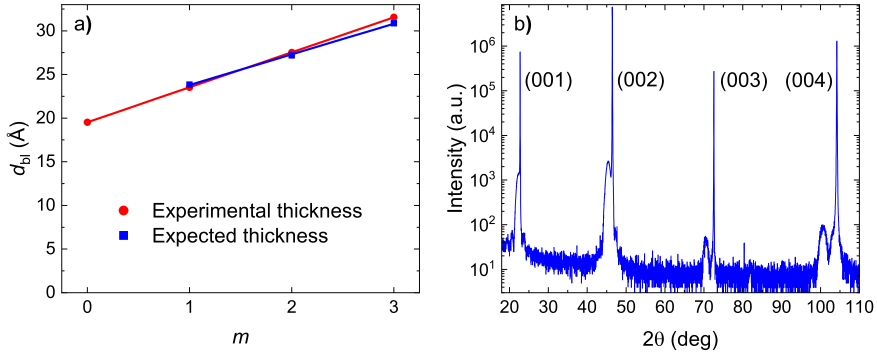


Supplementary Fig. 1: **Determinaton of bilayer thickness from X-ray measurements.** (a) Comparison of the thickness of $(LaFeO_3)_m$+$(SrTiO_3)_5$ bilayer, $d_{bl}$, determined from X-ray diffraction and the one expected from $SrTiO_3$ and $LaFeO_3$ lattice constants as detailed in the text. (b) X-ray diffraction scan of 8 nm thin $LaFeO_3$ film on (001) oriented $SrTiO_3$ substrate.

* kiaba@mail.muni.cz

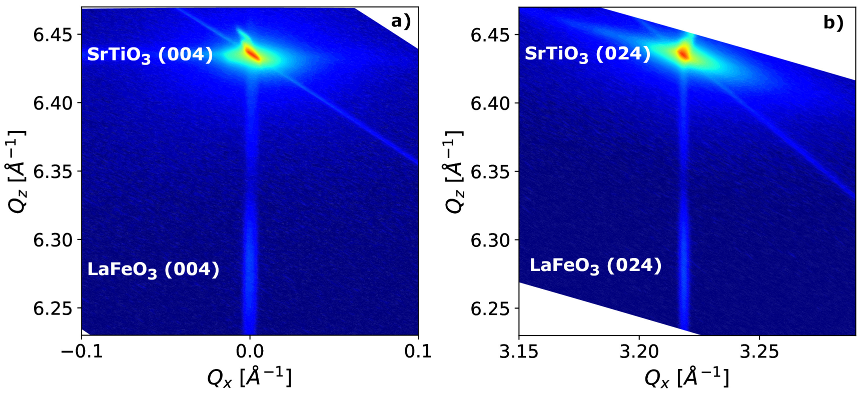


Supplementary Fig. 2: **Reciprocal space maps of 8 nm thin $LaFeO_3$ film near (a) (004) and (b) (024) $SrTiO_3$ diffraction.**

## II. LOW ENERGY MUON SPIN ROTATION

The low-energy muon spin rotation (LE$\mu$SR) spectroscopy [1, 2] with relatively small muon penetration depth compared to the standard muon spin rotation ($\mu$SR) enables studying thin films and heterostructures. We have used the muon beam with 2 keV that provides an implantation profile where most of the muons stop in the superlattices; see Supplementary Fig. 4(b). The implantation profile was calculated using Monte Carlo TRIM.SP code [3]. Data in all measurements are analyzed above 0.1 $\mu$s since below, artifacts due to back reflection of muons arise [2].

### A. Zero field muon spin rotation

The zero-field asymmetry spectra presented in Fig. 2(a)-(c) do not exhibit any oscillatory time evolution typically seen in bulk crystals. This arises because the structure of our superlattices leads to a broad distribution of internal fields, including the stray fields due to the iron spin canting that spread through $SrTiO_3$ layers. This corresponds to a large distribution of Larmor frequencies inevitably leading to a fast damping of the oscillations.

The time evolution of the zero-field asymmetry presented in Fig. 2(a)-(c) was fitted using the stretched exponential function (1) whose exponent $\beta$ and depolarization rate, $\lambda$, are is shown in Fig. 2(d) and (e) respectively. For completeness, the initial asymmetry, $A_0$, is shown in Supplementary Fig. 5. The temperature dependence of $A_0$ exhibits a noticeable decrease below $T_{\mathrm{N}}$, that is, below 175 K for the $m = 3$ and below 35 K for the $m = 2$ superlattice. This decrease is expected in an ordered magnetic phase where the muons quickly depolarize due to strong static local fields. In the $m = 1$ superlattice, $A_0$ exhibits only a gradual and relatively weak decrease with decreasing temperature without a sharper onset in agreement with the interpretation that there is no static order in this superlattice. Surprisingly, $A_0$ of the $m = 1$ superlattice seems to increase from 10 to 5 K, however, this increase is on the level of one standard deviation.

### B. Weak transverse field muon spin rotation

In the weak transverse field data analysis, we shall consider the formation of muonium (a bound state of a muon and an electron) in $SrTiO_3$. Because the muon spin in muonium precesses at a different frequency than a free muon spin, the formation of muonium occurring below about 50 K in $SrTiO_3$ [4] influences the weak transverse field data of our superlattices. The temperature dependence of the normalized weak transverse field asymmetry for $SrTiO_3$ at 1.6 keV implanting energy was described by the empiric equation [4]

$$A_{\mathrm{N}}(T) = 0.1 \arctan \frac{T - 43}{4.427} + 0.85 \,, \qquad (1)$$

see Supplementary Fig. 6. Our measurements were performed at the implanting muon energy of 2 keV, which is close enough to use Supplementary Eq. (1) as a starting point in muonium correction. Assuming that the muonium is formed only in $SrTiO_3$ layers, the depolarization due to the muonium formation is subtracted from the data using

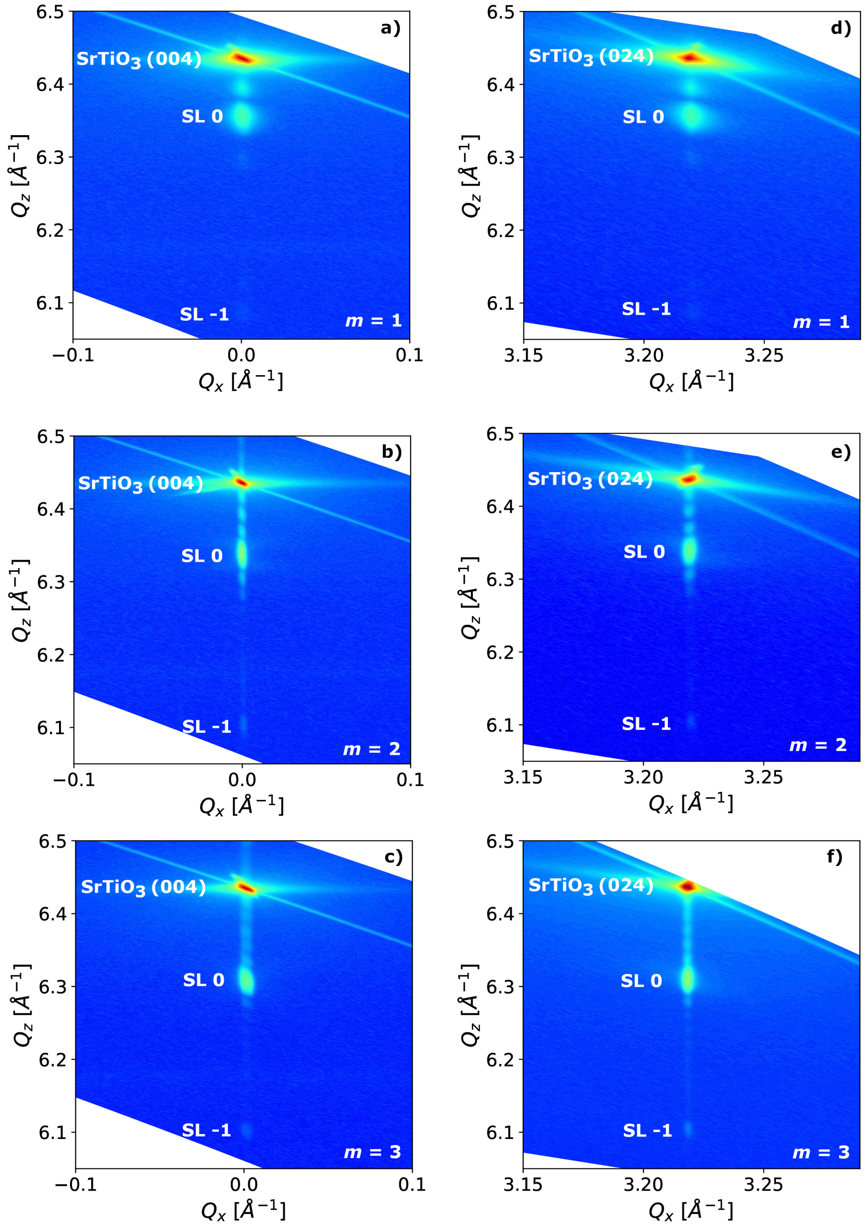


Supplementary Fig. 3: **Reciprocal space maps of the superlattices near the (004) and (024) diffractions of $SrTiO_3$ substrate.** Panels (a) and (d) are for the superlattice with $m = 1$, panels (b) and (e) are for the superlattice with $m = 2$, and panels (c) and (f) are for the superlattice with $m = 3$.

the following equation

$$A_{0,c}(T) = A_0(T) + f_{\mathrm{SrTiO_3}}\,[1 - A_{\mathrm{N}}(T)]\,A_0(T_{\mathrm{high}})\,, \qquad (2)$$

where $f_{\mathrm{SrTiO_3}}$ is the volume fraction of $SrTiO_3$ in a given superlattice, and $A_0(T_{\mathrm{high}})$ is the asymmetry at high enough temperature. For $A_0(T_{\mathrm{high}})$ we have used a mean value above 250 K where the superlattices are in the paramagnetic state and the influence of muonium is negligible. The correction for the muonium formation is significant only below the temperature of the muonium formation of about 50 K, where $A_{\mathrm{N}}(T)$ is significantly smaller than unity, see Supplementary Fig. 6. At higher temperatures, $A_{\mathrm{N}}(T) \approx 1$ and the second term on the right-hand side of Supplementary Eq. (2) vanishes.

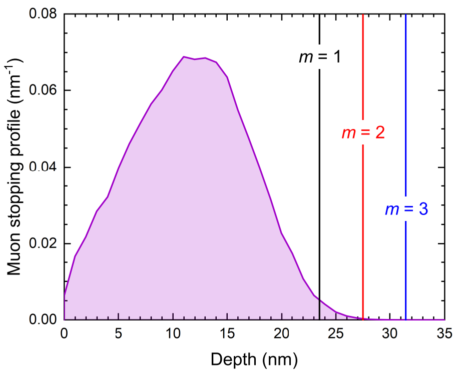


Supplementary Fig. 4: **Simulated implantation profile for a muon beam energy of 2 keV.** Vertical lines mark the thicknesses of $[(LaFeO_3)_m/(SrTiO_3)_5]_{10}$ superlattices.

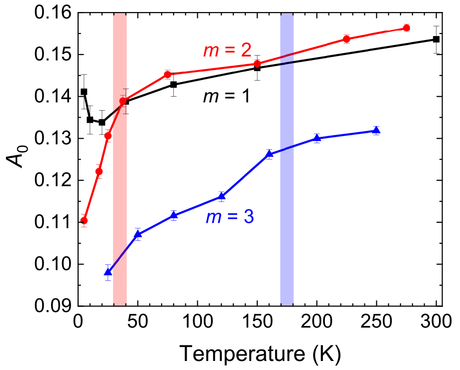


Supplementary Fig. 5: **Initial asymmetry, $A_0$, from the simulation of zero field measurement by the stretched exponential function.** The highlighted areas mark Neel temperature of $m = 2$ and $m = 3$ superlattices.

The magnetic volume fraction of the superlattice, $f_{\rm mag}(T)$, is calculated as [5]

$$f_{\rm mag}(T) = 1 - \frac{A_{0,c}(T)}{A_{0,c}(T_{\rm high})} \ , \tag{3}$$

where $A_{0,c}(T_{\rm high})$ is the mean of the initial weak transverse field asymmetry above 250 K in the expected paramagnetic state. Supplementary Fig. 7(a) shows $f_{\rm mag}$ calculated without the muonium correction (using $A_{\rm N}(T) = 1$). The shaded regions show temperatures below about 50 K where the muonium formation takes place. Values of $f_{\rm mag}$ corrected for the muonium formation using Supplementary Eq. (1), see Supplementary Fig. 7(b), suddenly decrease below about 50 K for superlattices with $m = 2$ and $m = 1$, which leads, for the case of $m = 1$, even to nonphysical values significantly below zero. Most likely, the step-like correction for muonium formation using Supplementary Eq. (1), which was obtained on $SrTiO_3$ single crystal, is sharper and centered at a different temperature than what would be appropriate for ultrathin $SrTiO_3$ layers of our superlattices. We have therefore adjusted the temperature and width of the transition in Supplementary Eq. (1) where the muonium formation occurs so that $f_{\rm mag}$ is not negative for the superlattice $m = 1$. This approach yielded

$$A_{N,\rm mod}(T) = 0.1 \arctan \frac{T - 20}{6} + 0.85 \ , \tag{4}$$

see Supplementary Fig. 6(b). Corresponding $f_{\rm mag}$ is shown in Supplementary Fig. 7(c) and in the main part of the paper in Fig. 3(b). Note that we did not adjust the multiplication factor of the step-like arctan function in Supplementary Eq. (4) corresponding to the magnitude of the correction. Consequently, the values of $f_{\rm mag}$ at 5 K resulting from the two corrections [cf. Supplementary Figs. 7(b) and 7(c)] are almost the same. Similarly, the main conclusions are the same: the magnetic volume fraction in the $m = 1$ superlattice at 5 K is essentially zero corresponding to the absence of a static order formed in the measured temperature range in contrast to the $m = 2$ superlattice where it is significantly above zero (above 0.4 and above $LaFeO_3$ volume fraction of 2/7) and thus the superlattice exhibits a static antiferromagnetic order.

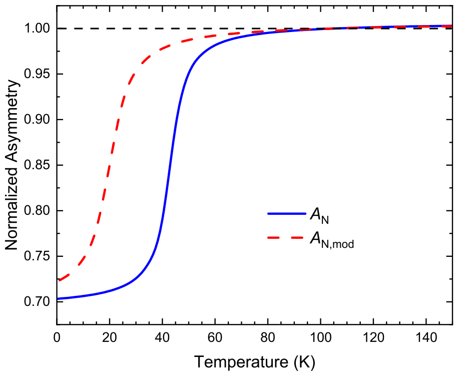


Supplementary Fig. 6: **Normalized asymmetry due to the muonium formation in $SrTiO_3$.** Normalized asymmetry expressed by Supplementary Eq. (1) is shown with blue solid line and by Supplementary Eq. (3) is shown with red dashed line.

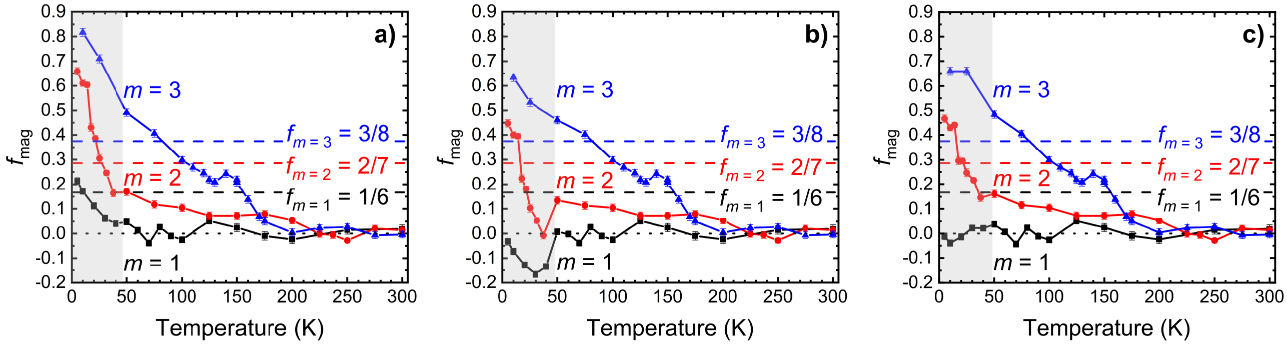


Supplementary Fig. 7: **Magnetic volume fractions obtained from the weak transverse field measurement.** Panel (a) shows values obtained without the correction for muonium formation, and panels (b) and (c) show those corrected for the muonium formation using Supplementary Eq. (1) and Supplementary Eq. (3), respectively.

### C. Longitudinal field muon spin rotation

Supplementary Fig. 8 shows the asymmetry of the $m = 1$ superlattice measured at 5 K for several longitudinal fields. The data are analyzed with the model

$$A_{\mathrm{LF}}(t, B_{\mathrm{ext}}) = A\left[c\, P_{\mathrm{dyn}}(t, B_{\mathrm{ext}}) + (1-c)P_{\mathrm{stat}}(t, B_{\mathrm{ext}})\right] + A_0(B_{\mathrm{ext}}) \; . \tag{5}$$

Here $A_0(B_{\mathrm{ext}})$ is a background asymmetry which, in principle, depends on magnetic field $B_{\mathrm{ext}}$. In LE$\mu$SR, muons are focused onto the sample by the external magnetic field, and thus different magnetic fields give rise to a different background. $A$ is the normalization constant that is field independent. The depolarization due to the sample is modeled as a weighted average of the theoretical Gaussian Kubo-Toyabe functions for the static disorder, $P_{\mathrm{stat}}$ [6],

$$P_{\mathrm{stat}}(B_{\mathrm{ext}} = 0, t) = \frac{1}{3} + \frac{2}{3}(1 - \sigma_{\mathrm{s}}^2 t^2)\exp\left[-\frac{\sigma_{\mathrm{s}}^2 t^2}{2}\right] \; , \tag{6}$$

$$\begin{aligned} P_{\mathrm{stat}}(B_{\mathrm{ext}}, t) = & 1 - \frac{2\sigma_{\mathrm{s}}^2}{(\gamma_\mu B_{\mathrm{ext}})^2}\left[1 - \exp\left(-\frac{\sigma_{\mathrm{s}}^2 t^2}{2}\right)\cos\left(\gamma_\mu B_{\mathrm{ext}} t\right)\right] + \\ & + \frac{2\sigma_{\mathrm{s}}^4}{(\gamma_\mu B_{\mathrm{ext}})^3}\int_0^t \exp\left(\frac{\sigma_{\mathrm{s}}^2 \tau^2}{2}\right)\sin\left(\gamma_\mu B_{\mathrm{ext}} \tau\right) d\tau \; , \end{aligned} \tag{7}$$

and the dynamic fluctuation, $P_{\mathrm{dyn}}$ [7],

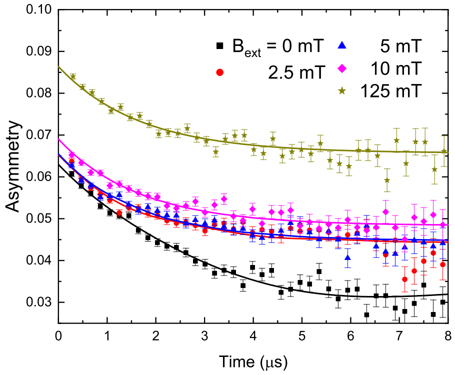


Supplementary Fig. 8: **Time evolution of the asymmetry from longitudinal field measurement of the $m = 1$ superlattice at 5 K.** Measured data for several applied magnetic fields is displayed together with a model (solid lines), see Supplementary Eq. (5). The error bars denote one standard deviation.

| Variable | Value |
|---|---|
| $\sigma_{\rm s}/\gamma_\mu$ [mT] | $0.32 \pm 0.08$ |
| $\sigma_{\rm d}/\gamma_\mu$ [mT] fixed | 250 |
| $\nu$ [GHz] | $150 \pm 20$ |
| $c$ | $0.64 \pm 0.06$ |
| $A$ | $0.032 \pm 0.003$ |
| $A_0(B_{\rm ext} = 0$ mT) | $0.030 \pm 0.002$ |
| $A_0(B_{\rm ext} = 2.5$ mT) | $0.032 \pm 0.003$ |
| $A_0(B_{\rm ext} = 5$ mT) | $0.032 \pm 0.003$ |
| $A_0(B_{\rm ext} = 10$ mT) | $0.036 \pm 0.003$ |
| $A_0(B_{\rm ext} = 125$ mT) | $0.053 \pm 0.003$ |

Supplementary Table I: Values of parameters obtained from the global fit of the longitudinal field data by Supplementary Eq. (5). The errors represent one standard deviation.

$$P_{\rm dyn}(B_{\rm ext}, t) = \exp\left[-\frac{2\sigma_{\rm d}^2\nu}{(\gamma_\mu B_{\rm ext})^2 + \nu^2}t\right], \qquad (8)$$

where $\nu$ is the fluctuation rate. The depolarization rate $\sigma$ appearing in Supplementary Eqs. (6)-(8) is defined as $\sigma = \gamma_\mu\sqrt{< \Delta B^2 >}$ where $< \Delta B^2 >$ is the second moment of the field distribution. The subscripts 's' and 'd' denote whether the second moment corresponds to the static or dynamic magnetic field distribution. The volume fraction of the dynamically fluctuating part is expressed by the field-independent parameter $c$.

The data shown in Supplementary Fig. 8 (points) were analyzed with a global fit of asymmetries at all measured fields with the model expressed by Supplementary Eq. (5) (solid lines). The obtained values of parameters are shown in Supplementary Tab. I. Because of the high correlation between $\sigma_{\rm dyn}$ and the fluctuation rate $\nu$, we fixed the distribution width of the dynamically fluctuating moments, $\sigma_{\rm dyn}/\gamma_\mu$, to 250 mT since similar values of the internal fields were observed in orthoferrites [8]. The corresponding value of the fluctuation rate is $\nu = 150 \pm 20$ GHz. Note that the fluctuation rate is unreasonably high, and a more detailed model involving a more realistic distribution of local magnetic fields yields a fluctuation rate about three orders of magnitude lower [9]. The obtained value $\sigma_{\rm s}/\gamma_\mu = 0.32 \pm 0.08$ mT exhibits a relatively large error that is due to the comparably large field of 2.5 mT used in the measurements. To determine this value with better precision, one would need to measure with significantly smaller fields. The errors of other values shown in Supplementary Tab. I are reasonably low (about 10%), which demonstrates that the global fit is well conditioned. Particularly, the fit allowed us to determine the constants $A$ and $A_0(B_{\rm ext})$ with reasonable precision. For the sake of simplicity, we display in Fig. 4 the data as normalized asymmetry

$$A^N_{\rm LF}(t, B_{\rm ext}) = [A_{\rm LF}(t, B_{\rm ext}) - A_0(B_{\rm ext})]/A\ . \qquad (9)$$

## III. RUN LOGS

| Measurement | $T$ [K] | $E$ [keV] | $B$ [mT] | run no. | year |
|---|---|---|---|---|---|
| Zero field | 300 | 1.96 | 0 | 4664 | 2022 |
| | 150 | 1.96 | 0 | 4693 | 2022 |
| | 80 | 1.96 | 0 | 4665 | 2022 |
| | 40 | 1.96 | 0 | 4666 | 2022 |
| | 20 | 1.96 | 0 | 4667 | 2022 |
| | 10 | 1.96 | 0 | 4668 | 2022 |
| | 5 | 1.96 | 0 | 4669 | 2022 |
| Weak transverse field | 300 | 2.01 | 10 | 6608 | 2021 |
| | 250 | 2.01 | 10 | 6609 | 2021 |
| | 200 | 2.01 | 10 | 6610 | 2021 |
| | 175 | 2.01 | 10 | 6611 | 2021 |
| | 150 | 2.01 | 10 | 6612 | 2021 |
| | 125 | 2.01 | 10 | 6613, 6607 | 2021 |
| | 100 | 2.01 | 10 | 6614, 6596 | 2021 |
| | 90 | 2.01 | 10 | 6615, 6597 | 2021 |
| | 80 | 2.01 | 10 | 6616, 6598 | 2021 |
| | 70 | 2.01 | 10 | 6617, 6599 | 2021 |
| | 60 | 2.01 | 10 | 6618, 6600 | 2021 |
| | 50 | 2.01 | 10 | 6619, 6601 | 2021 |
| | 40 | 2.01 | 10 | 6620, 6602 | 2021 |
| | 30 | 2.01 | 10 | 6621, 6603 | 2021 |
| | 20 | 2.01 | 10 | 6622, 6604 | 2021 |
| | 10 | 2.01 | 10 | 6623, 6605 | 2021 |
| | 5 | 2.01 | 10 | 6624, 6606 | 2021 |
| Longitudinal field | 5 | 1.96 | 0 | 4677, 4678 | 2022 |
| | 5 | 1.96 | 2.5 | 4679, 4680 | 2022 |
| | 5 | 1.96 | 5 | 4681, 4682 | 2022 |
| | 5 | 1.96 | 10 | 4683, 4684 | 2022 |
| | 5 | 1.96 | 125 | 4695, 4696 | 2022 |

Supplementary Table II: Low energy $\mu$SR run log for the $m = 1$ superlattice measured in zero, weak transverse, and longitudinal fields.

| Measurement | $T$ [K] | $E$ [keV] | $B$ [mT] | run no. | year |
|---|---|---|---|---|---|
| Zero field | 275 | 2.01 | 0 | 4833 | 2021 |
| | 225 | 2.01 | 0 | 4832 | 2021 |
| | 150 | 2.01 | 0 | 4831 | 2021 |
| | 75 | 2.01 | 0 | 4830 | 2021 |
| | 37.5 | 2.01 | 0 | 4829 | 2021 |
| | 25 | 2.01 | 0 | 4828 | 2021 |
| | 17.5 | 2.01 | 0 | 4826 | 2021 |
| | 5 | 2.01 | 0 | 4827 | 2021 |
| Weak transverse field | 300 | 2.01 | 10 | 4823 | 2021 |
| | 275 | 2.01 | 10 | 4822 | 2021 |
| | 250 | 2.01 | 10 | 4834 | 2021 |
| | 237 | 2.01 | 10 | 4821 | 2021 |
| | 225 | 2.01 | 10 | 4820 | 2021 |
| | 200 | 2.01 | 10 | 4819 | 2021 |
| | 175 | 2.01 | 10 | 4818 | 2021 |
| | 150 | 2.01 | 10 | 4817 | 2021 |
| | 125 | 2.01 | 10 | 4816 | 2021 |
| | 100 | 2.01 | 10 | 4815 | 2021 |
| | 75 | 2.01 | 10 | 4814 | 2021 |
| | 50 | 2.01 | 10 | 4813 | 2021 |
| | 37.5 | 2.01 | 10 | 4824 | 2021 |
| | 31.27 | 2.01 | 10 | 4835 | 2021 |
| | 25 | 2.01 | 10 | 4812 | 2021 |
| | 21.3 | 2.01 | 10 | 4836 | 2021 |
| | 17.5 | 2.01 | 10 | 4825 | 2021 |
| | 14 | 2.01 | 10 | 4837 | 2021 |
| | 10 | 2.01 | 10 | 4810 | 2021 |
| | 5 | 2.01 | 10 | 4811 | 2021 |

Supplementary Table III: Low energy $\mu$SR run log for the $m = 2$ superlattice measured in zero and weak transverse fields.

| Measurement | $T$ [K] | $E$ [keV] | $B$ [mT] | run no. | year |
|---|---|---|---|---|---|
| Zero field | 250 | 1.96 | 0 | 4158 | 2021 |
| | 200 | 1.96 | 0 | 4155 | 2021 |
| | 160 | 1.95 | 0 | 4156 | 2021 |
| | 120 | 1.95 | 0 | 4157 | 2021 |
| | 80 | 1.95 | 0 | 4152 | 2021 |
| | 50 | 1.96 | 0 | 4154 | 2021 |
| | 25 | 1.95 | 0 | 4153 | 2021 |
| Weak transverse field | 320 | 2.01 | 10 | 4140 | 2021 |
| | 300 | 2.01 | 10 | 4139 | 2021 |
| | 275 | 2.01 | 10 | 4138 | 2021 |
| | 250 | 2.01 | 10 | 4137 | 2021 |
| | 225 | 2.01 | 10 | 4136 | 2021 |
| | 200 | 2.01 | 10 | 4135 | 2021 |
| | 175 | 2.01 | 10 | 4134 | 2021 |
| | 170 | 2.01 | 10 | 4141 | 2021 |
| | 160 | 2.01 | 10 | 4142 | 2021 |
| | 150 | 2.01 | 10 | 4143,4133 | 2021 |
| | 140 | 2.01 | 10 | 4144 | 2021 |
| | 130 | 2.01 | 10 | 4145 | 2021 |
| | 125 | 2.01 | 10 | 4132 | 2021 |
| | 120 | 2.01 | 10 | 4146 | 2021 |
| | 110 | 2.01 | 10 | 4147 | 2021 |
| | 100 | 2.01 | 10 | 4131 | 2021 |
| | 75 | 2.01 | 10 | 4148 | 2021 |
| | 50 | 2.01 | 10 | 4149 | 2021 |
| | 25 | 2.01 | 10 | 4150 | 2021 |
| | 10 | 2.01 | 10 | 4151 | 2021 |

Supplementary Table IV: Low energy $\mu$SR run log for the $m = 3$ superlattice measured in zero and weak transverse fields.

[1] Prokscha, T. *et al.* The new $\mu$E4 beam at PSI: A hybrid-type large acceptance channel for the generation of a high intensity surface-muon beam. *Nucl. Instrum. Methods Phys. Res. A: Accel. Spectrom. Detect. Assoc. Equip.* **595**, 317–331 (2008). doi:10.1016/j.nima.2008.07.081

[2] Suter, A., Martins, M. M., Ni, X., Prokscha, T. & Salman, Z. Low energy measurements in Low-Energy $\mu$SR. *J. Phys.: Conf. Ser.* **2462**, 012011 (2023). doi:10.1088/1742-6596/2462/1/012011

[3] Eckstein, W. *Computer Simulation of Ion-Solid Interactions*, vol. 10 (Springer Berlin Heidelberg, 1991). doi:10.1007/978-3-642-73513-4

[4] Salman, Z. *et al.* Direct spectroscopic observation of a shallow hydrogenlike donor state in insulating $SrTiO_3$. *Phys. Rev. Lett.* **113**, 156801 (2014). doi:10.1103/PhysRevLett.113.156801

[5] Fowlie, J. *et al.* Intrinsic magnetism in superconducting infinite-layer nickelates. *Nat. Phys.* **18**, 1043–1047 (2022). doi:10.1038/s41567-022-01684-y

[6] Hayano, R. S. *et al.* Zero-and low-field spin relaxation studied by positive muons. *Phys. Rev. B* **20**, 850–859 (1979). doi:10.1103/PhysRevB.20.850

[7] Keren, A. Generalization of the abragam relaxation function to a longitudinal field. *Phys. Rev. B* **50**, 10039–10042 (1994). doi:10.1103/PhysRevB.50.10039

[8] Holzschuh, E., Denison, A. B., Kündig, W., Meier, P. F. & Patterson, B. D. Muon-spin-rotation experiments in orthoferrites. *Phys. Rev. B* **27**, 5294–5307 (1983). doi:10.1103/PhysRevB.27.5294

[9] Kiaba, M. Interface and doping induced phenomena in transition metal oxide heterostructures. Online. Doctoral theses, Dissertations. Brno: Masaryk University, Faculty of Science. Available from: `https://is.muni.cz/th/iy62v/?lang=en`.
